# Medication supply chain resilience during disasters: exploration of causes, strategies, and consequences applying Strauss and Corbin's approach to the grounded theory

**DOI:** 10.1186/s40545-023-00604-6

**Published:** 2023-08-10

**Authors:** Peivand Bastani, Omid Sadeghkhani, Parisa Bikine, Gholamhossein Mehralian, Mahnaz Samadbeik, Ramin Ravangard

**Affiliations:** 1 College of Health and Human Sciences, Charles Darwin University, Alice Springs, NT 0870 Australia; 2 School of Dentistry, UQ Oral Health Center, The University of Queensland, Brisbane, QLD 4006 Australia; 3 Student Research Committee, School of Health Management and Information Sciences, Shiraz University of Medical Science, Shiraz, Iran; 4 Nottingham Business School, Nottingham Trent University, Nottingham, UK; 5 Social Determinants of Health Research Center, Lorestan University of Medical Sciences, Khorramabad, Iran; 6 Health Human Resources Research Center, School of Health Management and Information Sciences, Shiraz University of Medical Science, Shiraz, Iran

**Keywords:** Resilience, Supply chain management, Disaster, Qualitative study, Grounded theory

## Abstract

**Background:**

Due to the importance of procurement of medicines and medical supplies during disasters and the significance of an existing resilient supply chain, the present study aimed to explore the causes, strategies, and consequences of the medication supply chain resilience during the Kermanshah, Iran, earthquake experience in 2018.

**Methods:**

This was a qualitative study based on the grounded theory method. The suggested approach by Strauss and Corbin was used. Thirty-two in-depth and semi-structured interviews were conducted via theoretical sampling, and data were saturated. Open-ended and probing questions were used, and all the interviews were recorded with the prior permission of the participants. Field notes and memoing were also used along with the interviews. Transcribed data were analyzed in three levels of open coding, selective, and axial coding by two researchers with sufficient reflexivity without any conflict of interest.

**Results:**

The results led to the identification of eight main categories, including "Disaster Management Structure", "Information Management", "Supply Chain Monitoring", "Socio-Cultural Factors", "Planning", "Resource Management", "Medical Service Coverage", and "Waste of time and resources". According to Strauss and Corbin's model, socio-cultural factors and inappropriate structures and planning related to the health system during disasters may waste time and resources.

**Conclusions:**

"Waste of time and resources” during disasters can be considered the main determinant that can damage the resilience of the medication supply chain. Policymakers need to seek applied strategies for decreasing waste. Socio-cultural interventions, preparedness of information infrastructures and coordination among the stewards and the community during disasters can help the supply chain preserve its resilience and act more effectively.

## Background

Medicines and medical supplies are considered among the most critical human health commodities and are vital for patients worldwide [1]. Medication unavailability adversely affects the community`s health [2]. The vital role of medicines and medical supplies is undeniable in improving the health services` functions at different levels, and the availability of medicines and medical supplies indicates the quality of health services [3]. Lack of medicines and medical supplies could be a more significant problem during disasters [4].

Disasters could affect predicting the amount of medicine and medical supplies and on-time procuring, particularly in developing settings [5]. While the provision of medicines and medical supplies is one of the critical priorities in developing countries [6], disruption in their supply, including the flow of medical and pharmaceutical supplies as a critical outcome, could be more highlighted during disasters [7]. From the managerial perspective, disruption of the medication supply chain can have serious consequences. Companies often lose revenue and market share when other supply chains are disrupted. In contrast, disruption in the medication supply chain can endanger many people's lives. For this reason, continuity of operations in health services and supply chains is vital. This becomes important when the community is involved in significant health problem, including an infectious disease (for example, respiratory pandemics, such as the flu, etc.), an industrial accident (such as the release of large amounts of toxic substances in urban areas, etc.), a natural disaster (including earthquake, flood, etc.), or a terrorist incident (e.g., bomb blast, war, etc.) [7].

The determinants that cause a medication supply chain to be disrupted are mainly related to product non-compliance, product shortages, poor performance, distribution/patient safety errors, and technological issues due to the lack of pharmacy stock[8]. In contrast, an efficient medication supply chain provides them with stakeholders in a reasonable and acceptable quality, in the shortest possible time, and at the optimal cost [9]. Such a chain should not only be able to deal with disasters but also should have a high degree of flexibility in returning to its original state or moving towards more favorable conditions [10].

As the evidence implies, some strategies facilitate the appropriate response by the health sectors as well as the medication supply chains during disasters, among which we can consider predicting dynamic demand, dynamic allocation of resources, preparing a position list of products with a short useful life, cooperating and sharing information, and developing business models to minimize counterfeiting [7]. Similarly, Chen et al. have emphasized that recovery strategies such as implementing dynamic operations could help improve the supply chain's effectiveness during disasters [11]. Attention to the effectiveness and competitive advantages of the distributor–retailer interface is also recommended as a supply chain management strategy [12]. The success of implementing the appropriate strategy depends on the underlying conditions of the health system and its ability to coordinate internally and externally and manage the medication supply chain from both aspects of the structure and the health information system [13].

The Kermanshah earthquake, a 7.3 magnitude which occurred in November 2018, led to the loss of about 700 people's lives and more than 10 thousand cases of serious injuries. Lessoned learned from this natural disaster emphasized that the vulnerability of local health facilities, including medicines and medical supplies, could affect the quantity and quality of health services [ref 21]. Considering the causes, strategies, and consequences of the medication supply chain resilience during disasters based on the local context could help provide more effective, on-time services. This study explores the causes, strategies, and consequences of the resilience of the medication supply chain during the Kermanshah, Iran, earthquake experience in 2018.

## Methods

This qualitative study applies the grounded theory approach. Strauss and Corbin's design (2008) was used to conduct the study after the experience of the Kermanshah earthquake, one of the most significant natural disasters in Iran, in 2018.

Strauss and Corbin argue that the goal of the grounded theory method is to theoretically discover and explain complete expressions and interpretations of specific phenomena using regular techniques and analytical procedures that assist the researcher in processing a true theory [14]. Based on this approach, an attempt is made to review and analyze a complex social phenomenon with its context and explain the main consequences and causes of the phenomenon, action-interaction strategies, and intervening conditions in a model. The study's main objective was to explore the causes, strategies, and consequences of the medication supply chain resilience during disasters based on the local Iranian context during the Kermanshah, Iran, earthquake experience in 2018.

### Study participants

In the approach of Strauss and Corbin, participants should be selected based on their experiences in the process under study[15]. Therefore, based on the nature of the process and the study's main purpose, the primary participants were selected from those involved in the medication supply chain with sufficient experience in natural disasters. The purposive sampling method was used to collect data, and then the theoretical sampling method was used as the study progressed.

Through purposive sampling, those individuals rich in information or items through which the researchers could gain good knowledge about the study's main purpose were selected. In theoretical sampling, unlike methods used with previous planning, the selection of each new participant depends on the data obtained from previous participants. At the beginning of the study, the researchers started sampling started sampling people who could provide the necessary information about the research topic and analyzed the initial information. After creating the initial codes, the researchers continued the sampling process with different people in various organizations through theoretical sampling to obtain the necessary concepts to create the model. Finally, 32 members of non-profit organizations, universities of medical sciences, military organizations, health insurance organizations, and pharmaceutical distribution companies were selected as the study participants, and the data reached theoretical saturation with this number. Table 1 indicates the details of these participants.

**Table 1:** Study participants` description

Participant	Age (year)	Gender	Education LEVEL	Work experience (year)
1	42	Male	PhD	15
2	49	Male	PhD	27
3	48	Male	MD/GP	22
4	44	Male	Diploma	10
5	33	Male	Bachelor	13
6	40	Male	MD/GP	20
7	49	Male	MD/GP	26
8	54	Male	PharmD	16
9	38	Male	PhD	19
10	40	Male	PharmD	4
11	32	Male	PharmD	16
12	44	Male	Master`s	21
13	39	Male	PhD	29
14	50	Male	Bachelor	12
15	37	Male	PharmD	10
16	36	Male	PharmD	14
17	39	Male	Dentist	20
18	45	Male	Master`s	24
19	48	Male	PhD	21
20	48	Male	Bachelor	22
21	43	Female	Bachelor	12
22	40	Male	MD/GP	22
23	43	Male	Bachelor	13
24	39	Male	Master`s	30
25	52	Male	MD/GP	16
26	44	Male	MD/GP	5
27	29	Male	Bachelor	20
28	52	Male	Bachelor	19
29	52	Male	MD/GP	20
30	47	Male	Bachelor/nurse	29
31	54	Male	Bachelor/nurse	13
32	43	Male	MD/GP	13

### Data collection

The required data were collected using in-depth interviews, observation, memoing, and field notes. This study's main data collection method was semi-structured in-depth interviews using open-ended questions. For this purpose, the researchers first communicated with the participants and then collected data by asking general and open-ended questions. The researchers also asked probing questions such as "Can you explain more?" or "Give an example?" to gather complete information on the research topic. All the interviews were recorded after prior permission; transcriptions were prepared word by word after several times listening to the interview audio files.

Corbin (2008) believes that in theoretical sampling, interview and observation guides do not have an organized and fixed structure as much as possible, because they are changed and completed during the research period. In the present study, the semi-structured interview guide started with general questions with the least likely structure, and more specific questions were asked based on the initial results and objectives of the study, according to the interviewees’ experiences and responses. In addition, simultaneously with the progress of research and the formation of emerging theories, changes were made in the interview guide.

Moreover, in this study, the researchers used memoing to record the ideas and relationships that came to their minds when collecting and analyzing data. Memoing is an essential part of the data analysis process, and its purpose is to keep ideas and thoughts in the bracket. Due to their abstract nature, these memos form the basic framework of the theory [14, 16, 17]. Field notes were another source of data collection for this study. These notes are the same as memos. Researchers used these field notes to be sure of documenting the observations and conversations in the field and their thoughts and experiences during their observation.

### Data analysis

In grounded theory studies, the constant data comparison method is used to analyze the data in which the data are collected, coded, and analyzed simultaneously. In this study, open coding, axial coding, and selective coding were used step by step to analyze the data. For open coding, the text of transcribed interviews and field notes were reviewed and re-read several times to examine the data line by line and word for word. The sentences and concepts in each line and paragraph were identified and coded. In axial coding, the researcher seeks to answer questions such as "Why?", "How did it happen?", "Where?", "How?", "When?" and "With what results?" and in response to these questions, the categories, and the relationship between them are identified. Some classes merge, or a new category is formed [18].

In selective coding, the researchers used the central category selection criteria proposed by Strauss and Corbin (2008) to find the central or core category and establish relationships among the concepts to relate to the emerging concepts. Criteria for selecting the central category include having the power of interpret of the analysis, having centrality, repetition of data, communication with other categories, justification of many differences between categories, and the emergence of maximum change and analysis [15].

### Data robustness

In the present study, four Lincoln and Guba criteria, including credibility, dependability, confirmability, and transferability, were used to ensure the robustness and trustworthiness of the data and results. In this regard, the researchers used the peer-checking method to provide the text of several interviews and their codes to several colleagues familiar with conducting qualitative studies to verify the trustworthiness of the coding process. Sampling with maximum diversity was also used as a transferability technique. Data source, method, and investigator triangulation were used to ensure the credibility of the data, and the results were reviewed by researchers who had no conflict of interest with the research topic. Furthermore, a long-term and continuous in-field observation technique was applied to increase the credibility, and one of the researchers (OS) was settled in the field during the data collection process.

### Ethical consideration

This study was approved by the Shiraz University of Medical Sciences Ethics Committee (Code: IR.SUMS.REC.1397.776). Oral and written informed consents were obtained from all the participants and they were assured that the information would be completely anonymous and confidential.

## Results

The results led to the identification of eight main categories. These categories include "Disaster Management Structure", "Information Management", "Supply Chain Monitoring", "Socio-Cultural Factors", "Planning", "Resource Management", "Medical Service Coverage", and "Waste of time and resources ".

**Fig. 1:**
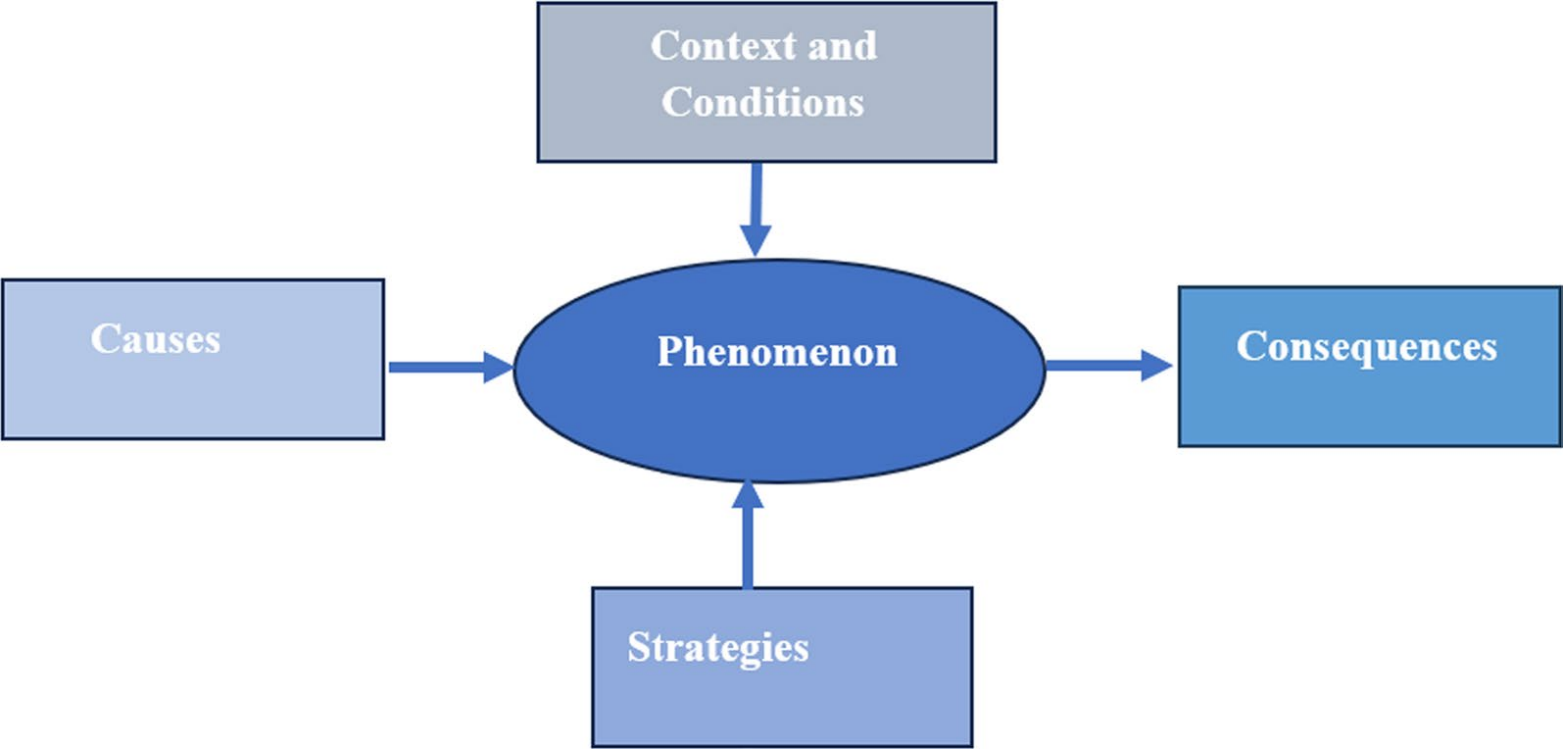
Coding paradigm of the study

As the main objective of this study was to explore the causes, strategies, and consequences of the medication supply chain resilience during disasters, Strauss and Corbin's coding paradigm (2008) was used for a better illustration and connecting the structural factors of the phenomenon to the process of health system resilience. This coding paradigm includes three main components, as follows (Fig. 1):

Elaborating Fig. 1,* Conditions or structures:* indicate the results from answering the following questions help the researchers determine the conditions: What? Why?, Where?, and How did it happen?

*Action/reaction responses:* Existing action–reaction/emotional response is rooted in situations, problems, and events.

*Consequences:* Consequences of the process occurs in response to action–reaction/emotional response to events.

**Fig. 2:**
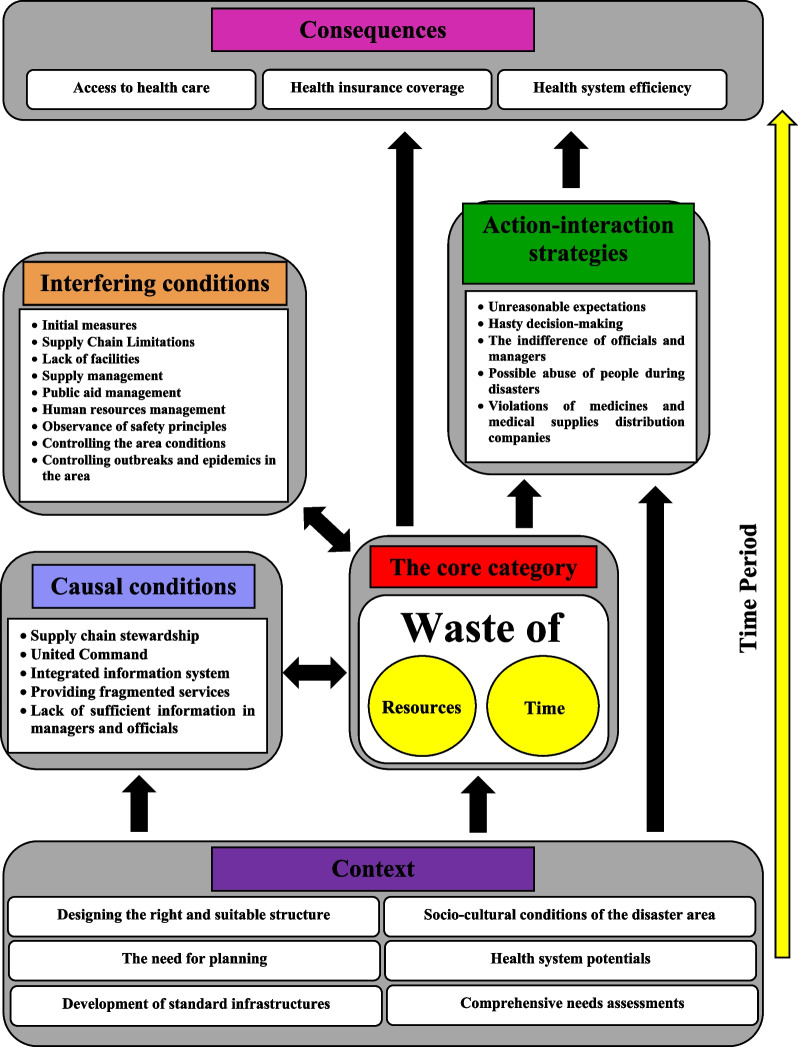
Causes, strategies, and consequences of medication supply chain resilience during disasters

The narrative story for this coding paradigm is illustrated in Fig. 2. Figure 2 shows that “the waste of time and resources” has been identified as the main category or the core of the model presented for causes, strategies, and consequences of medication supply chain resilience during disasters. Accordingly, unclear stewardship of the medication supply chain, lack of a united command in the health sector and lack of inter-sectoral coordination with other institutions and organizations involved, lack of an integrated information system, lack of accurate, up-to-date, and sufficient information for decision making, and the provision of fragmented and non-goal-based services can lead to the waste of time and resources during disasters. The lack of proper management of resources and their waste can lead to limited access to pharmaceutical and medical services for people in crisis and reduce the efficiency of the healthcare system. In addition, health insurance coverage is also damaged due to the lack of an integrated information system, lack of comprehensive needs assessment, and lack of proper information flow between service providers and service users during disasters. In this process, underlying factors such as socio-cultural conditions, characteristics, and potentials of the health sector, the need for designing an appropriate structure and effective planning, comprehensive needs assessment and the development of infrastructure standards will also be influential.

**Fig. 3:**
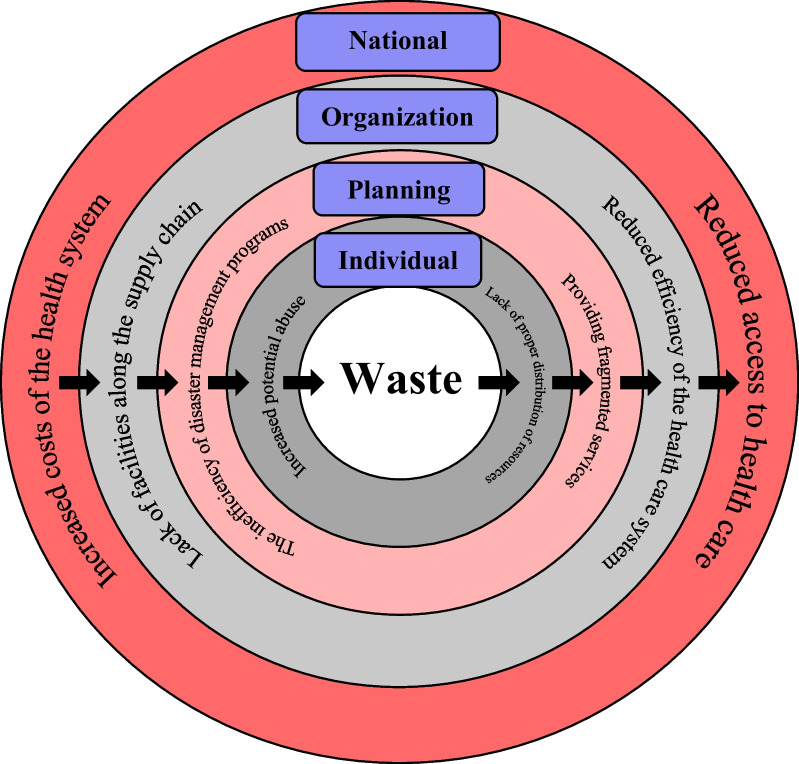
The conditions–outcomes matrix

Although Fig. 2 tries to identify the key points, provides the relationship between the structure and process, and helps understand the structure, to strengthen the analysis of the relationship between the main categories, the conditions–outcomes matrix was also illustrated (Fig. 3). This matrix reinforces the analysis by categorizing and organizing the conditions/outcomes. At the same time, the matrix helps to better understand the process by showing the relationship between macro conditions and the dynamics and changes in action/interaction conditions and results.

Considering both figures, the causes, strategies, and consequences of the medication supply chain resilience during disasters can be described narratively as follows:

Following the exposure of the health care system to waste, the lack of resources and facilities will occur in the disaster area and, due to the lack of infrastructural and structural readiness to deal with the disaster, the system will inevitably use the resources available in the medical centers of the region and centers near the disaster site. The use of the resources of the medical centers close to the disaster site disrupts in the provision of medical services in these centers, and its consequence is a disruption in the medicines and medical supplies distribution chain during disasters. Due to the presence of various organizations in disaster management and the overlap of their tasks, the weakness in united command and management causes duplication and provision of fragmented services in the region because of which the severity of waste increases. At the same time, poor information coordination among organizations involved in disaster management prevents comprehensive need assessment in the region. As a result, a shortage or surplus of resources will be found in the region, leading to abuse or violation. Therefore, strengthening the healthcare infrastructure to prevent the destruction of medical centers in the region and providing medical services in the standard hospital can prevent chaos and disorder in the region.

Moreover, in the case of formulating operational plans, proper management of resources, and appropriate use of potentials of the health care system, the amount of waste and loss can be significantly reduced. In contrast, organizations need more participation and coordination in designing and developing joint scenarios to deal with the disaster, reducing organizational capacity. It increases duplication in the medication supply chain. In such an environment, poor condition of the medication supply chain and lack of sufficient information at the management and official leaders` level may easily lead to hasty decision-making and a sense of indifference against critical situations, which result in negative consequences for medical care coverage.

In sum, the healthcare network contains important field information for local distribution and prevention of the occurrence and spread of various diseases during disasters. However, there are also structural and planning weaknesses in critical situations. Waste of time and resources during disasters causes weaknesses of health insurance organizations in creating health insurance coverage in the disaster area. Experienced people, relying on the lessons learned from past disasters, could make the right decisions, and take the necessary measures to prevent the waste of time and resources in the supply chain of medicines and medical supplies.

## Discussion

The results of the present study show that "waste of time and resources” is the most critical category in the medication supply chain`s resilience during disasters. As Sazvar et al. mentioned, serious attention to the identification and classification of pharmaceutical wastes cannot only save money and reduce the costs in a supply chain, manage production time, generate additional revenue, and decrease the environmental impacts [19]. In addition, strategies to reduce inventory waste as the most valuable physical resource, along with managing human resources and other tangible and non-tangible resources, should be considered for increasing the resilience of a pharmaceutical supply chain [20]. Similarly, results of a review showed that five types of challenges should be considered during natural disasters, including identifying uncertainty, mismatch between plans and reality, lack of establishment of a crisis organization, need to adapt medical and pharmaceutical responses with the actual needs and ensure resilient response. This result is significant due to their customization with the Middle East context as Iran belongs to [21].

Other results of the present study show that sociocultural factors and the need for planning and designing an appropriate disaster management structure are among the most important underlying factors that can affect the prevention of waste of time and resources during disasters. While "waste of time and resources" has been recognized as the core category, finding the influential and underlying factors before any applied intervention would be sensible. Based on the results of Coperich et al. paying attention to underlying factors such as the optimal time of medication supply and the level of inventory supply can lead to minimal costs and reduced waste of resources during disasters [22]. Supply chain agility is another strategy that has been proposed as a response to high levels of supply chain complexity and uncertainty [23] and can be considered during disasters. Mehralian et al. believe that achieving an agile supply chain depends on various factors, including supply chain capabilities, such as flexibility, responsiveness, technical competence, and speed [24]. Components of delivery speed, planning, trust development, supplier evaluation and prioritization, environmental pressure, performance management, and information technology tools are among other strategies for developing an agile supply chain [25]. These factors can be changed or become unstable in critical situations, making supply chain resilience difficult during disasters. Therefore, disaster planning and attention to passive defense programs in this field are becoming increasingly necessary.

According to the present results, resource and information management were two other important factors identified as practical strategies to increase medication supply chain resilience. With an integrated, accurate, valid, and up-to-date information system, resource management and waste reduction will be possible during disasters. In this regard, the study of Rasheed et al. shows that the existence of an up-to-date information system for determining epidemiological needs and medicine requirements in the disaster area can be a vital factor in quantifying and estimating required pharmaceutical items and ordering them through the supply chain [26]. Attempts to make and update surveillance systems and need-based decision support systems can be helpful in this area.

Other results of the present study indicate that there needs to be more efficiency in disaster management planning, along with the lack of possible facilities in the medication supply chain during disasters can intensify the waste of time and resources and incur additional costs for the health system. This category is essential in getting accurate information about common diseases and people's medical needs for routine medicines and determining emergency needs resulting from disasters. Altiok and Melamed have concluded that the urgency of unpredictable pharmaceutical needs during natural disasters is not comparable to the routine pharmaceutical needs of the region in terms of quantity, and the lack of proper forecasting mechanisms can be considered one of the most important challenges experienced in these times [27]. Similarly, results from the same earthquake in Iran indicating challenges in relief operations show that logistic challenges, including need assessment, procurement, warehousing, transportation, and distribution, are among the central areas of significance in this earthquake experience. Lessons from this study and similar ones emphasized the need to understand better the challenges involved in disaster relief operations conducted by multiple actors and help them improve their practices, creating proper regulations, policies, and logistics strategies [28].

Regarding the results of the current study, performing a comprehensive needs assessment and designing an appropriate structure, along with paying attention to socio-cultural factors of the region, are among other important factors that can prevent the waste of pharmaceutical resources during disasters. In their study in Finland, Jahre and Heigh showed that establishing local relief infrastructure was the best way to deal with critical situations [29]. Haavisto et al. also emphasize that spending $ 1 in the infrastructure preparation and design phase equals $ 3 in the disaster response phase [30]. Such fundamental infrastructures should be recognized based on the context, and further actions should be implemented to prepare, set up, and keep them upgraded and applicable. In other words, having comprehensive needs assessment program based on the region's characteristics, including its epidemiological needs, disease, and demographic characteristics, can increase supply chain resilience and reduce resource waste both before and during disasters and thus can help increase the efficiency of the health system during disasters.

Based on social and cultural factors, as shown by the results of Kasdan’s study, the role of society and people's behavior according to their social and cultural characteristics during disasters can be beneficial or destructive [31]. Therefore, the government should pay attention to these factors and develop localized policies under cultural conditions, social needs, and the level of acceptance and cooperation of the people of each region. In addition to strengthening the regional collective spirit, advocacy, and cooperation, this can help use the capacity of local infrastructure and prevent waste of resources and facilities. In addition, paying attention to coping strategies may help create a resilient supply chain during disasters. These strategies can be aimed at society and the people, as well as the managers, officials, those involved in providing services, and finally, the companies that produce, import, and distribute pharmaceutical and medical products. Having a strategic view of the disaster phenomenon, especially natural disasters, using interactive approaches between stakeholders and community representatives can help make these strategies more operational and ultimately prevent the waste of time and resources during disasters.

### Strengths

One of the main strengths of the present study from the methodology perspective was that the researchers at the beginning and all stages of the research used the idea reflexivity by rethinking their thoughts, ideas, and feelings about the topic under study and became aware of and put them in writing. In addition, a summary of the results, along with the main categories, sub-categories, and the central concept, was provided to the participants to be reviewed and ensure the trustworthiness of the results.

### Limitations

This study has some restrictions. Strauss and Corbin’s model may lead to a structured generation of the concepts. This can be moderated by applying open coding processes and combining observation, document analysis, and in-depth interviews. In addition, the small sample size and the possibility of participant bias can be among other methodological limitations. Further areas of research could be recommended in this area, applying the mix-method approach to estimate the potential resilience level of the medication supply chain in Iran and a comprehensive understanding of the factors that affect this resilience during disasters.

## Conclusions

"Waste of time and resources” during disasters can be considered the main determinant that can damage the resilience of the medication supply chain. This can lead to negative consequences such as inefficiencies in the whole health system, health service access, and health insurance coverage challenges. To achieve resilience in the medication supply chain during disasters at all organizational, local, and national levels, health policymakers must seek applied and context-based strategies for decreasing the waste of resources. Sociocultural interventions, preparing necessary information infrastructures and providing better coordination among the stewards and the community during disasters are recommended as practical strategies to improve the resilience of the medication supply chain. Improving logistic operations, monitoring the coordination between the local need assessments and the pharmaceutical supplies and procurement along with the transparency of information flow and trust building in the vulnerable community could be among some practical recommendations for improving medication supply chain resilience.

## Data Availability

The data sets used and/or analyzed during the current study are available from the corresponding author on reasonable request.
